# Clinical Aspects of Neonatal Hypoglycemia: A Mini Review

**DOI:** 10.3389/fped.2020.562251

**Published:** 2021-01-08

**Authors:** Taygen Edwards, Jane E. Harding

**Affiliations:** Liggins Institute, University of Auckland, Auckland, New Zealand

**Keywords:** newborn, insulin, screening, diagnosis, continuous glucose monitoring, oral dextrose gel, child development

## Abstract

**Introduction:** Neonatal hypoglycemia is common and a preventable cause of brain damage. The goal of management is to prevent or minimize brain injury. The purpose of this mini review is to summarize recent advances and current thinking around clinical aspects of transient neonatal hypoglycemia.

**Results:** The groups of babies at highest risk of hypoglycemia are well defined. However, the optimal frequency and duration of screening for hypoglycemia, as well as the threshold at which treatment would prevent brain injury, remains uncertain. Continuous interstitial glucose monitoring in a research setting provides useful information about glycemic control, including the duration, frequency, and severity of hypoglycemia. However, it remains unknown whether continuous monitoring is associated with clinical benefits or harms. Oral dextrose gel is increasingly being recommended as a first-line treatment for neonatal hypoglycemia. There is some evidence that even transient and clinically undetected episodes of neonatal hypoglycemia are associated with adverse sequelae, suggesting that prophylaxis should also be considered. Mild transient hypoglycemia is not associated with neurodevelopmental impairment at preschool ages, but is associated with low visual motor and executive function, and with neurodevelopmental impairment and poor literacy and mathematics achievement in later childhood.

**Conclusion:** Our current management of neonatal hypoglycemia lacks a reliable evidence base. Randomized trials are required to assess the effects of different prophylactic and treatment strategies, but need to be adequately powered to assess outcomes at least to school age.

## Introduction

Neonatal hypoglycemia is a preventable cause of brain injury. It is common, affecting 5–15% of all babies (1) and approximately half of at-risk babies (2) and is associated with a range of adverse sequelae (3, 4). However, the optimal frequency and duration of screening, as well as the threshold at which treatment would prevent brain injury, remains uncertain. The purpose of this review is to summarize the recent advances in clinical aspects of transient neonatal hypoglycemia.

## Pathophysiology of Neonatal Hypoglycemia

Glucose is the primary metabolic fuel for the fetus. The fetus receives glucose from its mother through carrier-mediated diffusion down a concentration gradient across the placenta (5, 6). Fetal glucose concentrations are ~80% of maternal concentrations and fluctuate with changes in maternal glucose concentrations (7). The function of insulin in the fetus is as a growth hormone rather than to regulate glucose concentrations, and secretion of insulin occurs at a lower glucose concentration in the fetus than in postnatal life (8).

Maternal and therefore fetal glucose concentrations increase during labor and delivery in response to secretion of maternal stress hormones such as catecholamines and glucocorticoids (9). Once the umbilical cord is clamped, glucose supply is interrupted and neonatal glucose concentrations decrease, reaching a low point ~1–2 h after birth. In turn, insulin secretion decreases while secretion of counter-regulatory hormones such as glucagon and catecholamines increases, stimulating gluconeogenesis and glycogenolysis, and resulting in a gradual increase in glucose concentrations (9). However, these do not reach adult concentrations until after 72 h of age (10, 11). Delay or interruption of this postnatal metabolic adaptation results in neonatal hypoglycemia.

Glucose is an essential metabolic fuel for the brain, and in the newborn the proportionately large brain accounts for almost all of total tissue glucose requirements (12). Thus, low glucose concentrations are likely to result in inadequate brain energy supply. Although the newborn brain can use alternative metabolic substrates, the supply of these is limited. Lactate provides a potential alternative fuel in the first 48 h, and ketones may be available on days 3–4, but each can provide only a small proportion of total brain energy requirements (13).

## Defining Neonatal Hypoglycemia

The definition of neonatal hypoglycemia remains controversial, and has changed over time (14). However, since the major reason for defining hypoglycemia is to identify a threshold at which treatment would prevent brain injury, an ideal definition would relate to the glucose concentration at which brain function is compromised. This makes a single definition problematic, as the threshold is likely to vary in different babies, depending amongst other things on gestational age, postnatal age, concurrent metabolic demands, co-morbidities and availability of alternative metabolic fuels.

The most widely used definition for neonatal hypoglycemia is a glucose concentration of <47 mg/dl (2.6 mmol/l) (15–17). This arises primarily from two studies published in 1988, which related glucose concentrations to neurological function. One was a retrospective study of 661 preterm babies (birthweight <1,850 g), which reported that a glucose concentration of <47 mg/dl (2.6 mmol/l) on three or more days was associated with an increased risk of developmental delay at 18 months' corrected age (18). Follow-up of a subgroup showed that reduced motor and arithmetic functioning persisted at 8 years (19).

The second study recorded brainstem or somatosensory evoked potentials in 17 infants, of whom only five were newborns (20). None showed flattening of evoked potentials with a glucose concentration of >47 mg/dl (2.6 mmol/l), although some with a glucose concentration below this still had normal evoked potentials. Both studies concluded that a glucose concentration of >47 mg/dl (2.6 mmol/l) was likely to be safe.

In situations where evidence-based decisions are not possible, operational thresholds offer a pragmatic guide to clinicians for when intervention may be warranted (1). Screening protocols have recommended different operational thresholds ranging from 18 to 60 mg/dl (1.0–3.3 mmol/l) (21–24). However, most recommend aiming for a minimum glucose concentration close to 47 mg/dl (2.6 mmol/l) in late preterm and term babies more than a few hours old or requiring treatment.

## Incidence and Risk Factors

The incidence of neonatal hypoglycemia varies between studies depending on the diagnostic threshold, the glucose screening protocol and measurement method used, and the population studied (25). However, the incidence of transient neonatal hypoglycemia is estimated to be 5–15% of newborns (1, 26), and in at-risk babies, it approximates 50% (2) (Table 1). Babies with multiple risk factors do not have a higher incidence but may experience more severe hypoglycemia.

**Table 1:** Risk factors for neonatal hypoglycemia.

Transient neonatal hypoglycemia
Preterm birth
Small or large for dates
Infant of diabetic mother
Perinatal stress (birth asphyxia, hypothermia, respiratory distress, sepsis)
Birth asphyxia
Poor feeding
Maternal use of beta blockers
Antenatal corticosteroids
Persistent neonatal hypoglycemia^*^
Congenital hyperinsulinism
Hypopituitarism (ACTH deficiency, growth hormone deficiency)
Cortisol deficiency
Glycogen storage disease
Disorders of gluconeogenesis (FBP deficiency, PEPCK deficiency, PC deficiency)
Fatty acid oxidation defects

* *Occurring after or persisting for ≥3 days (27)*.

*ACTH, Adrenocorticotropic hormone; FBP, Fructose-1,6-bisphophatase; PEPCK, Phosphoenolpyruvate carboxykinase; PC, Pyruvate carboxylase*.

## Management of Neonatal Hypoglycemia

### Screening for Neonatal Hypoglycemia

The clinical signs of neonatal hypoglycemia include, but are not limited to, cyanosis, apnea, altered level of consciousness, seizures, lethargy, and poor feeding (24). However, since many of these signs are non-specific, and the majority of babies with low glucose concentrations show no clinical signs, it is recommended that all babies with risk factors undergo regular glucose monitoring.

The optimal frequency and duration of screening remain uncertain. Most protocols recommend screening within 1–4 h after birth and then every 3 or 4 h until euglycemia is maintained over two or three consecutive glucose measurements (15, 21, 22, 24). However, all of these guidelines are informed by expert opinion and lack a reliable evidence base (28).

Some specify different monitoring periods dependent on the clinical profile of the baby. For example, the American Academy of Pediatrics recommends that monitoring continues until 12 h after birth for infants of diabetic mothers or large for gestational age, but for 24 h for babies who are born late preterm or small for gestational age (21). However, there is no evidence to suggest that cerebral glucose requirements vary between at-risk groups (15).

One study that screened at-risk babies using an accurate glucose oxidase method 1–2 h after birth then every 3–4 h before feeds for the first 24 h and every 3–8 h from 24 to 48 h reported no difference between risk groups in the incidence or severity of neonatal hypoglycemia, suggesting that a single screening protocol would be reasonable for all babies at risk (2).

### Blood Glucose Monitoring

#### Intermittent Glucose Monitoring

A common method for measuring glucose concentrations in neonates is by heel-prick blood sampling analyzed using point-of-care non-enzymatic glucometers. These provide quick results at a low cost, are readily available in neonatal units, user-friendly and require small volumes of blood (29).

However, these devices are designed for monitoring high glucose concentrations in diabetics, and are affected by several factors that vary widely in newborns including bilirubin concentrations and hematocrit. They are inaccurate at low glucose concentrations, with estimated false positive and false negative rates of 10–30%, and are not recommended as the sole method for diagnosis of neonatal hypoglycemia (21, 30). If point-of-care non-enzymatic glucometers are used for screening, it is critical to confirm the results with a laboratory method (21), but best practice is to use more accurate methods from the start.

Laboratory methods use enzymatic reactions including glucose oxidase, hexokinase or dehydrogenase (29) which are more accurate and sensitive for detecting neonatal hypoglycemia (31, 32). However, laboratory methods are costly, take time which can delay prompt intervention, and accuracy is also reliant on the quality of the plasma sample (29). More recent guidelines recommend blood gas analyzers which are quick and accurate if they are immediately available (15, 24).

A more feasible alternative in many settings is the newer enzymatic point-of-care analyzers, which have the same accuracy as laboratory methods but the convenience and speed of a cot-side measurement. Although they are more expensive per test than the widely used (but inaccurate) test strip glucometers, a recent cost analysis concluded that enzymatic glucometers incurred lower direct costs overall because they avoided the additional costs of retesting in the laboratory (33).

#### Continuous Interstitial Glucose Monitoring

Continuous interstitial glucose monitors comprise a sensor placed under the skin, and a recording device, often remote from the sensor, which converts the electrical current generated in the sensor to a glucose concentration using an inbuilt algorithm. Most devices provide a reading every 5 min, giving detailed information about glycemic control including the duration, frequency, and severity of hypoglycemia (34).

Continuous glucose monitors have several limitations. They require calibration against blood glucose concentrations at least every 12 h, so they do not abolish the need for blood tests, and more frequent calibration is recommended for greater accuracy and precision (35). Continuous glucose monitors are also prone to measurement error, and the reading can drift from the calibrated value without detection (35). Because, like point-of-care glucometers, they are designed for use in diabetes, they are less accurate at low glucose concentrations. The lag period between changes in blood glucose concentrations and changes in the continuous monitor reading is unknown but could be up to 30 min or more, due both to the time required for glucose to diffuse from blood to interstitial fluid, and to delays built into the algorithms, so that the rapid changes in glucose concentrations that are common in newborn babies are poorly reported by continuous monitors (36, 37). Infection at the site of sensor insertion is a theoretical concern, but in practice has rarely been reported, and most studies have reported that sensors can be left in place for a week without complications, even in very low birthweight babies (38).

Most importantly, there is a lack of evidence on whether continuous glucose monitoring is associated with clinical benefits or harms. Continuous glucose monitoring detects many more episodes of low glucose concentrations than does intermittent blood glucose measurement. For example, in 102 babies at risk of hypoglycemia, continuous glucose monitoring identified 11% more babies and 50% more episodes of low glucose than intermittent glucose monitoring (39). Others have reported similar differences (38, 40). Thus, there is a risk that continuous glucose monitoring may lead to a large increase in diagnosis and treatment, but without evidence that these additional detected episodes are related to brain injury, or that additional treatment will have any long-term benefit.

Despite these limitations, continuous glucose monitoring has enormous potential to improve the management of neonatal hypoglycemia. A randomized trial in 48 very low birthweight babies showed that use of continuous glucose monitoring reduced the number of blood samples taken, detected more episodes of neonatal hypoglycemia and reduced the duration of an episode by half when compared with intermittent glucose monitoring (40). Another randomized trial in 50 very preterm babes reported that continuous glucose monitoring in conjunction with an algorithm for glucose infusion titration reduced the duration and severity of hypoglycemic episodes, thereby promoting glycemic stability (41). However, it is not yet known if this improved stability will lead to improved later outcomes.

### Treating Neonatal Hypoglycemia

The goal of treating neonatal hypoglycemia is to prevent or minimize brain injury by maintaining a glucose concentration above an acceptable threshold (25). The usual initial approach is to feed the baby, using either formula or breast milk. When glucose concentrations are <18–25 mg/dl (1.0–1.4 mmol/l) intravenous dextrose (bolus 200 mg/kg followed by an infusion of around 4–8 mg/kg per minute) is usually required (21, 24). However, administering intravenous dextrose involves admission to the neonatal intensive care unit (NICU), which is costly, invasive, and separates the mother from her baby, which in turn can increase maternal anxiety and interfere with the establishment of breastfeeding. Severe or prolonged hypoglycemia, indicated by persistently high or ongoing (≥3 days) intravenous glucose requirements, suggest underlying endocrine or metabolic pathology and further investigation is required (Table 1). Elevated insulin concentrations indicate hyperinsulinism, which suppresses the production of alternative metabolic fuels, and hence maintaining blood glucose ≥ 3.5 mmol/l is recommended (24). Additional treatments, such as diazoxide (42), glucagon (24, 43) or glucocorticoids (44) may be required.

Oral dextrose gel 200 mg/kg (0.5 ml/kg of 40% dextrose), in combination with feeding, is increasingly recommended as a first-line treatment for asymptomatic neonatal hypoglycemia (45, 46). A randomized trial of 237 late preterm and term babies at risk of neonatal hypoglycemia [ <47 mg/dl (2.6 mmol/l)] demonstrated that compared with feeding alone, 40% oral dextrose gel 200 mg/kg plus feeding resulted in fewer treatment failures (hypoglycemia after two treatment attempts), reduced admission to NICU for hypoglycemia and reduced formula feeding at 2 weeks of age (47). A 2-year follow-up established safety by demonstrating similar rates of processing difficulty and neurosensory impairment between the oral dextrose and placebo groups (48). A subsequent cost-utility analysis concluded that dextrose gel resulted in a cost-saving of US$782 per baby (49).

The incorporation of oral dextrose gel into clinical practice has been evaluated in pre-and post-introduction observational studies in several parts of the world, with most reporting that oral dextrose was associated with a reduced NICU admission and increased breastfeeding (50–54). Its use is now recommended in several national guidelines (15, 22, 24).

### Prophylaxis

There is some evidence that even transient and undetected episodes of neonatal hypoglycemia may be associated with adverse sequelae. One study of 1,395 babies born in a center where glucose screening was universal showed that a single episode of transient neonatal hypoglycemia [ <35 mg/dl (1.9 mmol/l)] was associated with lower 4th-grade literacy and numeracy proficiency at 10 years of age (55). The Children With Hypoglycemia and Their Later Development (CHYLD) study demonstrated that clinically undetected low interstitial glucose concentrations were associated with an increased risk of executive dysfunction at 4.5 years of age (56). These findings suggest that even an effective treatment for neonatal hypoglycemia would not be sufficient to optimize outcomes for all babies, and prophylaxis needs to be considered.

The prophylactic measures currently recommended include early feeding, ensuring babies are warm and dry, and early skin-to-skin contact (57). These measures are thought to have a glucose sparing effect (58), but the evidence that they alter blood glucose concentrations or the incidence of hypoglycemia is limited (59–61).

Oral dextrose gel is being tested as an additional prophylactic measure to prevent hypoglycemia in at-risk babies. A dose-finding trial (Pre-hPOD) of 416 at-risk babies randomized to either placebo or dextrose gel at one of four different dosing schedules reported that a single dose of prophylactic oral 40% dextrose gel (200 mg/kg) in combination with breastfeeding was the most effective and practical dose (62), with a number needed to treat to prevent one case of hypoglycemia of 10. Further, the treatment was found to be acceptable, well tolerated, and had no adverse events (62). Follow-up at 2 years' corrected age showed no adverse effects, similar rates of neurosensory impairment between the groups, and a trend toward improved executive function scores in the dextrose gel group (63).

A quasi-experimental study of 236 at-risk babies reported that compared with feeding, prophylactic oral dextrose gel 200 mg/kg was not associated with a decreased incidence of hypoglycemia [ <40 mg/dl (2.2 mmol/l)] or admission to NICU (64). However, this study was not randomized, and the preparation used (Insta-Glucose gel) includes additional carbohydrates other than dextrose, which are likely to have competed with dextrose for membrane uptake and potentially reduced the effectiveness of this approach.

A multicenter randomized trial (hPOD) investigating whether prophylactic oral dextrose gel prevents neonatal hypoglycemia and hence reduces NICU admission has finished recruitment (ANZC Trials Registry – ACTRN12614001263684) (65). The results, and particularly the findings of the planned long-term follow-up, will provide valuable insight into whether prophylaxis with dextrose gel should be introduced into clinical practice.

## Outcomes of Neonatal Hypoglycemia

Magnetic Resonance Imaging (MRI) studies have shown that neonatal hypoglycemia can cause brain injury (66, 67). The most widely reported pattern of acute brain injury is localized in the parietal and occipital regions (68), which are involved in visual processing. However, the evidence is inconsistent on whether neonatal hypoglycemia is associated with later visual problems (69). Injury may extend beyond these regions with reports of global or periventricular damage (67) as well as damage to the basal ganglia and thalamic regions (67, 70).

A systematic review and meta-analysis of six cohort studies with a sample size of 1,675 babies reported that neonatal hypoglycemia [definitions ranged from <20–47 mg/dl (1.1–2.6 mmol/l)] was not associated with neurodevelopmental impairment, cognitive or motor deficits between 2 and 5 years of age (4). However, neonatal hypoglycemia was associated with a 3-fold increased risk of visual-motor impairment and executive dysfunction at 4 years of age. These risks were heightened for children who had experienced severe, recurrent or clinically undetected neonatal hypoglycemia (56). In older children, limited data (two studies, sample size of 54 babies) showed that neonatal hypoglycemia was associated with more than a 3-fold increased risk of neurodevelopmental impairment at 6–11 years of age, and a 2-fold increase in low numeracy and literacy (4). No studies reported on outcomes for adolescents.

Most of the evidence about long-term outcomes after neonatal hypoglycemia comes from retrospective observational studies, few of which have controlled for potential confounders or looked at outcomes beyond very early childhood. For example, infants of mothers with diabetes, who are at increased risk of neonatal hypoglycemia, have an increased risk of adverse outcomes (71, 72), but it is unclear how much of this risk is attributable to neonatal hypoglycemia. There is high heterogeneity between the studies which made comparing outcomes problematic, and there have been frequent calls for robust randomized trial evidence (3).

A randomized non-inferiority trial was the first to begin to address this major knowledge gap by comparing treatment at a threshold of 47 mg/dl (2.6 mmol/l) against treatment at a lower threshold of 36 mg/dl (2.0 mmol/l) among a sample of 689 otherwise healthy late preterm and term babies with mild-moderate hypoglycemia [36 mg/dl (2.0 mmol/l)−46 mg/dl (2.5 mmol/l)] (73). Babies with early (birth to 2 h) and severe [ ≤ 35 mg/dl (1.9 mmol/l)] hypoglycemia were excluded. In babies randomized to treatment at the lower threshold, fewer were monitored and treated, but there were more severe and recurrent hypoglycemic episodes (≥4 episodes) compared with babies in the higher threshold group. Hospital costs and duration of stay were similar between the groups, as were motor and cognitive functioning at 18 months on the Bayley Scales for Infant and Toddler Development. However, since previous studies have shown no relationship between neonatal hypoglycemia and motor or cognitive function at this age (4), this finding is not surprising, and the greater exposure to severe and recurrent hypoglycemia in the low threshold group is of concern. Much longer follow-up, at least to school age, will be essential to realize the true value of this important study.

## Conclusion

Over the last few years, neonatal hypoglycemia has received much attention. However, what remains unclear is the extent to which transient asymptomatic neonatal hypoglycemia is associated with brain injury and neurodevelopmental impairment, and if so, at what glucose concentration maintained for how long. To address this, adequately powered randomized trials are needed of both prophylactic and treatment interventions at different glucose thresholds, with neurodevelopmental outcomes assessed at least to school age.

## Author Contributions

TE and JH contributed to the literature search and the design of the review. TE wrote the first draft of the manuscript and contributed to further revisions. JH contributed to the writing of the manuscript and supervised the study. Both authors contributed to the article and approved the submitted version.

## Conflict of Interest

The authors declare that the research was conducted in the absence of any commercial or financial relationships that could be construed as a potential conflict of interest.
